# Association between *Lactobacillus *species and bacterial vaginosis-related bacteria, and bacterial vaginosis scores in pregnant Japanese women

**DOI:** 10.1186/1471-2334-7-128

**Published:** 2007-11-07

**Authors:** Renuka Tamrakar, Takashi Yamada, Itsuko Furuta, Kazutoshi Cho, Mamoru Morikawa, Hideto Yamada, Noriaki Sakuragi, Hisanori Minakami

**Affiliations:** 1 Department of Obstetrics and Gynecology, Hokkaido University Graduate School of Medicine, Kita-ku N14 W6, Sapporo, Japan, 060-8638

## Abstract

**Background:**

Bacterial vaginosis (BV), the etiology of which is still uncertain, increases the risk of preterm birth. Recent PCR-based studies suggested that BV is associated with complex vaginal bacterial communities, including many newly recognized bacterial species in non-pregnant women.

**Methods:**

To examine whether these bacteria are also involved in BV in pregnant Japanese women, vaginal fluid samples were taken from 132 women, classified as normal (n = 98), intermediate (n = 21), or BV (n = 13) using the Nugent gram stain criteria, and studied. DNA extracted from these samples was analyzed for bacterial sequences of any *Lactobacillus*, four *Lactobacillus *species, and four BV-related bacteria by PCR with primers for 16S ribosomal DNA including a universal *Lactobacillus *primer, *Lactobacillus *species-specific primers for *L. crispatus*, *L. jensenii*, *L. gasseri*, and *L. iners*, and BV-related bacterium-specific primers for BVAB2, *Megasphaera*, *Leptotrichia*, and *Eggerthella*-like bacterium.

**Results:**

The prevalences of *L. crispatus*, *L. jensenii*, and *L. gasseri *were significantly higher, while those of BVAB2, *Megasphaera*, *Leptotrichia*, and *Eggerthella*-like bacterium were significantly lower in the normal group than in the BV group. Unlike other *Lactobacillus *species, the prevalence of *L. iners *did not differ between the three groups and women with *L. iners *were significantly more likely to have BVAB2, *Megasphaera, Leptotrichia*, and *Eggerthella*-like bacterium. Linear regression analysis revealed associations of BVAB2 and *Megasphaera *with Nugent score, and multivariate regression analyses suggested a close relationship between *Eggerthella*-like bacterium and BV.

**Conclusion:**

The BV-related bacteria, including BVAB2, *Megasphaera*, *Leptotrichia*, and *Eggerthella*-like bacterium, are common in the vagina of pregnant Japanese women with BV. The presence of *L. iners *may be correlated with vaginal colonization by these BV-related bacteria.

## Background

Bacterial vaginosis (BV) is the disturbed vaginal flora, in which normal lactobacilli are replaced by an overgrowth of various anaerobic bacteria [1]. This condition is common in women of reproductive age [1,2] and may cause malodorous vaginal discharge, although in many women it is asymptomatic [3]. In pregnant women, bacterial vaginosis has been suggested to be a risk factor of perinatal complications, including preterm birth [1,4-12] and chorioamnionitis [4,13]. These complications are closely associated with neonatal morbidity and mortality worldwide.

Bacteria detected in BV flora include *Gardnerella vaginalis*, *Mycoplasma hominis*, *Mobiluncus *species (sp.), and other anaerobic bacteria, *i.e*., *Peptostreptococcus *sp., *Prevotella *sp., and *Bacteroides *sp. [1,14-16]. Recently, bacteria such as *Atopobium vaginae*, *Megasphaera *sp.*, Leptotrichia *sp., and *Eggerthella*-like bacterium have been reported as microorganisms related to this condition by molecular analyses [17-19]. Fredricks *et al*. identified three phylogenetically distinct bacterial DNA sequences in human vaginal samples highly specific for this condition and designated them BV-associated bacteria (BVAB) 1~3 [19]. They showed that BVABs, *Megasphaera*, *Leptotrichia*, and *Eggerthella*-like bacterium are more specific for BV than *Gardnerella *and *Atopobium *[19]. Among BVABs, BVAB2 was shown to be more sensitive for BV than BVAB1 and BVAB3, while the specificities of three BVABs were similar [19]. We use the term "BV-related bacteria" for bacteria including BVAB2, *Megasphaera*, *Leptotrichia*, and *Eggerthella*-like bacterium in this manuscript. However, it is important to note that these organisms have not been proven to be causative agents of BV.

The healthy human vaginal flora in reproductive age is usually predominated by *Lactobacillus *species. Their metabolic products, such as hydrogen peroxide (H_2_O_2_), lactic acid, and bacteriocin are believed to play an important role in maintenance of the normal vaginal flora by inhibiting colonization by other pathogens [20-23]. The predominant *Lactobacillus *species in the normal lactobacillary flora were shown by molecular biological analyses to be *L. crispatus*, *L. gasseri*, and *L. jensenii *[23-28]. In recent studies, *L. iners *described as L. 1086V by Antonio *et al*. [24] was identified as one of the common *Lactobacillus *species colonizing the human vagina [18,28-31]. Only 9% of the strains of this species produce H_2_O_2_, whereas almost all strains of *L. crispatus *and *L. jensenii *produce H_2_O_2 _[24].

To date, there have been few studies regarding the frequencies of the BV-related bacteria described above and *Lactobacillus *species in healthy and abnormal vaginal flora in pregnant women. The present study was performed to evaluate the prevalence of the BV-related bacteria and the common *Lactobacillus *species in normal and BV flora in pregnant Japanese women. We used a specific PCR method targeting the bacterial 16S ribosomal DNA (rDNA) region for this purpose.

## Methods

### Patients

A total of 163 pregnant Japanese women were enrolled in this study during routine prenatal visits at Hokkaido University Hospital from May 2005 to February 2006. Informed consent was obtained from all participants in verbal form. Vaginal fluid samples were collected at a mean of 23 weeks of gestation. Estimated date of delivery was determined from the last menstrual period and early gestational fetal ultrasonographic measurements.

### Sample collection, Nugent's scoring, and bacterial culture

A sterile speculum was inserted into the vagina and a specimen of vaginal fluid was obtained by brushing the posterior vaginal fornix with a swab. A vaginal smear was prepared by rolling a swab onto a glass slide, which was then air-dried, heat-fixed, and Gram-stained. The smears were then assessed according to Nugent criteria [32]. The other swab was spread onto Columbia blood agar plates, and incubated at 35°C under aerobic conditions in 5% CO_2 _and anaerobic conditions for 48 h. Lactobacilli were identified to the genus level by Gram staining of colonies and from colony morphology on blood agar plates.

### DNA extraction and PCR

Another swab was placed in 1 ml of PBS with subsequent vigorous vortexing to dislodge cells. The cells were centrifuged at 14,000 rpm for 5 min. The pellet was digested with proteinase K at 56°C for 30–60 min and the DNA was extracted and purified with a QIAmp DNA Mini Kit (Qiagen, Germantown, MD) in accordance with the manufacturer's instructions, resulting in 200 *μ*l of DNA solution. PCR mixtures consisted of PCR buffer with 1.5 mM of MgCl_2_, 10 pmol of each primer, 2.0 *μ*M of each deoxyribonucleoside triphosphate, 0.1 *μ*l of *Taq *DNA polymerase, and 1.5 *μ*l of template DNA solution in a final volume of 15 *μ*l.

Sequences and annealing temperatures for the various primer sets are listed in Table 1[19,33]. All primers were located in the 16S rDNA region. PCR was carried out for 40 cycles. For the *Lactobacillus *genus and its four species, the denaturation was performed at 95°C for 15 s followed by a 1-min annealing and extension step. For four BV-related bacteria, the denaturation step was set at 94°C for 30 s, followed by the annealing step for 30 s for BVAB2, *Megasphaera*, and *Leptotrichia *and for 40 s for *Eggerthella*-like bacterium, with extension at 72°C for 1 min for all reactions. A final extension step at 72°C for 7 min was added for all reactions. Aliquots of 7 *μ*l of the PCR products were electrophoresed in agarose gels and visualized by ultraviolet transillumination after ethidium bromide staining.

**Table 1:** PCR primers

Name	Sequence(5'-3')	Target	Annealing temperature (°C)	Reference
LactoF LactoR	TGGAAACAGRTGCTAATACCG GTCCATTGTGGAAGATTCCC	Lactobacillus	62	[33]
LcrisF LcrisR	AGCGAGCGGAACTAACAGATTTAC AGCTGATCATGCGATCTGCTT	*L. crispatus*	65	[33]
LjensF LjensR	AAGTCGAGCGAGCTTGCCTATAGA CTTCTTTCATGCGAAAGTAGC	*L. jensenii*	60	the present study
LgassF LgassR	AGCGAGCTTGCCTAGATGAATTTG TCTTTTAAACTCTAGACATGCGTC	*L. gasseri*	63	the present study
LinersF LinersR	CTCTGCCTTGAAGATCGGAGTGC ACAGTTGATAGGCATCATCTG	*L. iners*	65	the present study
Uncxb2-619F Uncxb2-1024R	TTAACCTTGGGGTTCATTACAA AATTCAGTCTCCTGAATCGTCAGA	BVAB2	55	[19]
Egger-621F Egger-859R	AACCTCGAGCCGGGTTCC TCGGCACGGAAGATGTAATCT	*Eggerthella*-like bacterium	58	[19]
Lepto-395F Lepto-646R	CAATTCTGTGTGTGTGAAGAAG ACAGTTTTGTAGGCAAGCCTAT	*Leptotrichia*	55	[19]
MegaE-456F MegaE-667R	GATGCCAACAGTATCCGTCCG CCTCTCCGACACTCAAGTTCGA	*Megasphaera*	55	[19]

The specificity of the *Lactobacillus *species-specific PCR for 14 common intestinal *Lactobacillus *species was evaluated and confirmed using 10^6 ^copies of one *Lactobacillus *species to each reaction as template DNA (Table 2). The universal *Lactobacillus *primer amplified all of these *Lactobacillus *species. The specific primers for *L. crispatus*, *L. jensenii*, and *L. gasseri *only amplified the corresponding species and did not amplify 13 other species (Table 2). They also did not amplify a cloned fragment of 16S rDNA region of *L. iners*. The specific primers for *L. iners *did not amplify any of 14 *Lactobacillus *species (Table 2). We analyzed PCR products from several vaginal samples amplified by the specific primers for *L. iners *and confirmed that the sequences of PCR products were completely consistent with *L. iners *(GenBank AY526083).

**Table 2:** Bacterial strains and the specificity of primers

Species	Strain	LactoF	LcrisF	LjensF	LgassF	LinersF
		LactoR	LcrisR	LjensR	LgassR	LinersR
*L. crispatus*	ATCC33197	+	+	-	-	-
*L. jensenii*	ATCC25258	+	-	+	-	-
*L. gasseri*	ATCC 4963	+	-	-	+	-
*L. acidophilus*	ATCC 4356	+	-	-	-	-
*L. brevis*	ATCC 14869	+	-	-	-	-
*L. casei*	ATCC 334	+	-	-	-	-
*L. delbrueckii*	ATCC 11842	+	-	-	-	-
*L. fermentum*	ATCC 14931	+	-	-	-	-
*L. johnsonii*	ATCC 11506	+	-	-	-	-
*L. helveticus*	ATCC 521	+	-	-	-	-
*L. plantarum*	ATCC 8014	+	-	-	-	-
*L. reuteri*	JCM1112	+	-	-	-	-
*L. rhamnosus*	ATCC 7469	+	-	-	-	-
*L. salvarius*	ATCC 11741	+	-	-	-	-

The sensitivities of the species-specific PCR for *L. crispatus*, *L. jensenii*, and *L. gasseri *were measured using serial dilutions of DNA solution of the reference strain. Similarly, for *L. iners*, serial dilutions of a cloned fragment of 16S rDNA region of *L. iners *were used instead. The sensitivity of the species-specific PCR for *L. crispatus*, *L. jensenii*, and *L. gasseri *and that of L. iners PCR were 10^2 ^to 10^3 ^copies and 10^2 ^copies per reaction, respectively.

### Statistical analysis

Fisher's exact probability test was used for statistical analysis. Multivariate logistic-regression analysis using SPSS™ for Windows was performed to evaluate the independent risk factors, and *P *< 0.05 was considered statistically significant.

## Results

### Clinical characteristics of women in three groups divided by Nugent score

A total of 163 samples from 163 pregnant women were obtained during the study period and 31 samples were excluded because of lack of information regarding the gestational week at delivery (n = 21) or Gram staining (n = 10). The remaining 132 samples from 132 women were analyzed and classified according to the Nugent criteria. Ninety-eight women (74.2%) were classified as having normal vaginal flora, 21 (15.9%) were intermediate, and 13 (9.8%) were BV. Samples from these women were divided into normal, intermediate, and BV groups, respectively.

The clinical characteristics of the pregnant women are summarized in Table 3. There were no statistically significant differences in the mean maternal age, number of nulliparous women, gestational week at sampling, gestational week at delivery, or birth weight of the neonate among the three groups. Of the total of 132 women, 35 (26.5%) delivered at <37 weeks, 9 (6.8%) at <33 weeks, and 4 (3.0%) at <30 weeks of gestation.

**Table 3:** Demographic and obstetric characteristics of women in normal, intermediate, and BV groups

	Nugent score					
	
	0–3		4–6		7–10	
No. of women	98		21		13	
Age (years)	32.6 ± 5.3	(19–44)	32.1 ± 5.8	(20–40)	29.1 ± 5.3	(21–37)
Nulliparity (%)	46.2		52.4		50.0	
Gestational week at sampling	22.6 ± 8.6	(5–36)	23.0 ± 9.1	(7–36)	24.2 ± 11.2	(7–37)
Gestational week at delivery	37.1 ± 2.9	(21–41)	36.4 ± 4.7	(18–40)	37.3 ± 5.5	(20–41)
Preterm birth at <37 weeks	26	26.5%	7	33.3%	2	15.4%
Preterm birth at <33 weeks	6	6.1%	2	9.5%	1	7.7%
Preterm birth at <30 weeks	2	2.0%	1	4.8%	1	7.7%
Birth weight (g)	2807 ± 565	(360–3805)	2602 ± 794	(165–3660)	2807 ± 831	(350–3555)

Range is shown in parenthesis.

### Detection rate of lactobacilli and BV-related bacteria in three groups by PCR

Genus *Lactobacillus *(any *Lactobacillus*) was detected in almost all women irrespective of Nugent score (Table 4). The detection rates of *L. crispatus*, *L. jensenii*, and *L. gasseri *were significantly higher in the normal group than in the BV group, while that of *L. iners *did not differ between the three groups. In contrast, the detection rates of BVAB2, *Megasphaera*, *Leptotrichia*, and *Eggerthella*-like bacterium were significantly lower in the normal group than in the BV group.

**Table 4:** Distribution of lactobacilli and bacterial vaginosis-related bacteria in women in normal, intermediate, and BV groups determined by PCR

	Nugent score		
	
	0–3	4–6	7–10
No. of women	98	21	13
any Lactobacillus	97(99.0%)	21(100.0%)	12(92.3%)
*L. crispatus*	60(61.2%)	6(28.6%)§	2(15.4%)§
*L. jensenii*	29(29.6%)	4(19.0%)	0(0.0%)†
*L. gasseri*	33(33.7%)	9(42.9%)	0(0.0%)§
*L. iners*	39(39.8%)	10(47.6%)	6(46.2%)
BVAB2	3(3.1%)	4(19.0%)†	5(38.5%)§
*Megasphaera*	11(11.2%)	13(61.9%)§	9(69.2%)§
*Leptotrichia*	14(14.3%)	5(23.8%)	7(53.8%)§
*Eggerthella*-like bacterium	7(7.1%)	7(33.3%)§	7(53.8%)§

†: P < 0.05, §: P < 0.01, vs group of Nugent score 0–3

### Independent risk factors for abnormal Nugent score

Multivariate logistic regression analysis was performed to evaluate the independent contributions of the various bacteria to the abnormal vaginal flora (Tables 5 and 6). Seven bacteria, *i.e*., *L. crispatus*,*L jensenii*,*L. gasseri*, BVAB2, *Megasphaera*,*Leptotrichia *and, *Eggerthella*-like bacterium, were entered as variates to be analyzed (*P *< 0.1, Fisher's exact probability test). The absence of *L. crispatus *and the presence of *Megasphaera *were selected as two independent risk factors of Nugent score ≥ 4, giving Odds ratios of 0.2 and 13.3, respectively (Table 5). Likewise, the presence of *Eggerthella*-like bacterium was selected as an independent risk factor of Nugent score ≥ 7, giving an Odds ratio of 6.2 (Table 6). Linear regression analyses revealed that BVAB2 and *Megasphaera *were associated with Nugent score.

**Table 5:** Independent risk factors for Nugent score ≥ 4 by multivariate regression analysis

	*β*	SE	p-value	Odds (95%CI)
Constant	-1.32	0.35		
*L. crispatus*	-1.51	0.52	0.004	0.22 (0.08–0.61)
*Megasphaera*	2.60	0.51	0.001	13.33 (4.92–36.11)

**Table 6:** Independent risk factors for Nugent score ≥ 7 by multivariate regression analysis

	*β*	SE	p-value	Odds (95%CI)
Constant	-2.24	0.47		
*Eggerthella*-like bacterium	1.83	0.65	0.005	6.24 (1.75–22.21)

### Coexistence of BV-related bacteria with *L. iners*

*L. iners *was detected by PCR in 55 of 132 (41.7%) women, and its prevalence did not differ between the groups classified according to Nugent score (Table 4). However, the presence of *L. iners *appeared to be positively associated with colonization by BV-related bacteria (Table 7). The detection rates of all BV-related bacteria were significantly higher in samples harboring *L. iners*. No such association was seen between the presence or absence of *L. iners *and the detection rate of any other *Lactobacillus *species.

**Table 7:** Prevalence of various bacteria according to the presence or absence of *L. iners*

	*L. iners*		
	
	present	absent	p-value
Number	55	77	
Lactobacillus	55 (100.0%)	75 (97.4%)	0.510
*L.crispatus*	25 (45.5%)	43(55.8%)	0.290
*L. jensenii*	13 (23.6%)	20(26.0%)	0.840
*L. gasseri*	15 (27.3%)	27(35.1%)	0.449
BVAB2	11 (20.0%)	1 (1.3%)	<0.001
*Megasphaera*	21 (38.2%)	12(15.6%)	0.004
*Leptotrichia*	19 (34.5%)	7(9.1%)	<0.001
*Eggerthella*-like bacterium	15 (27.3%)	6 (7.8%)	0.004

### Difference in the detection of *Lactobacillus *species between PCR and culture methods

*Lactobacillus *was cultured from 91 (92.9%) of 98 samples, 11 (52.4%) of 21 samples, and 2 (15.4%) of 13 samples of the normal, intermediate, and BV groups, respectively (data not shown). These observations conflicted markedly with the results obtained by the PCR method, especially in women with abnormal vaginal flora with respect to detection of *Lactobacillus*. Of the eleven women with BV from whom *Lactobacillus *was uncultured but detected by PCR, *L. iners *was detected in 5 women and *L. crispatus *was detected in only one woman by PCR, suggesting that *L. iners *is less likely to be cultured than *L. crispatus*. To determine which species of *Lactobacillus *is difficult to culture, the detection rates by PCR of various species of *Lactobacillus *were compared with those by the conventional culture method (Table 8). Among 130 samples determined to contain any *Lactobacillus *by the PCR method, 104 (80.0%) were positive for *Lactobacillus *by the culture method (Table 8). More than 90% of samples determined to contain *L. crispatus*, *L. jensenii*, or *L. gasseri *by the PCR method were determined to have *Lactobacillus *by the conventional culture method. Among 24 samples in which *L. crispatus *was the only *Lactobacillus *species identified by the PCR method, *Lactobacillus *was cultured from 22 samples (91.7%), while *Lactobacillus *was cultured only from 47.6% and 27.3% of samples in which *L. iners *and unspecified *Lactobacillus *species, respectively, were the only *Lactobacillus *species identified by the PCR method. Thus, *L. iners*, and unspecified *Lactobacillus *species other than *L. crispatus*, *L. jensenii*, or *L. gasseri *appeared to have stringent cultivation requirements.

**Table 8:** Differences in detection between PCR and cultivation methods

	PCR	CULTURE	CULTURE/PCR(%)
any Lactobacillus	130	104	80.0%
*L. crispatus*	68	63	92.6%
*L. jensenii*	33	32	97.0%
*L. gasseri*	42	39	92.9%
*L. iners*	55	41	74.5%
*L. crispatus *only	24	22	91.7%§
*L. jensenii *only	5	4	80.0%§
*L. gasseri *only	14	13	92.9%§
*L. iners *only	21	10	47.6%§
Lactobacillus, unspecified spp.only	11	3	27.3%§

§P < 0.01

Number of samples in which lactobacillus species were detected.

## Discussion

In the present study, we confirmed that *L. crispatus*, *L. gasseri*, and *L. jensenii *were common species in pregnant Japanese women with normal vaginal flora by species-specific PCR of the 16S rDNA region. These three species were less prevalent in women with BV. In contrast, four BV-related bacteria, *i.e*., BVAB2, *Megasphaera*, *Leptotrichia*, and *Eggerthella*-like bacterium, were detected at higher prevalence in women with BV. As all these results were in accordance with those of Fredricks *et al*. [19] who analyzed the vaginal fluid of non-pregnant women with and without BV using the broad-range 16S rDNA PCR and cloning methods, BV is suggested to have remarkably similar microbiological profiles among women with different demographic characteristics, including race and pregnancy, as suggested by the conventional cultivation method.

*L. crispatus*, *L. gasseri*, and *L. jensenii *are common *Lactobacillus *species found in the vagina [24-28,31,34]. *L. iners*, described recently as a new *Lactobacillus *species [29], is one of the common *Lactobacillus *species of the vaginal microbiota [18,19,23,28,30,31], which was also confirmed in the present study. The results showed that *L. iners *was present in 40% to 50% of women irrespective of Nugent score, as observed in an earlier study [19]. We examined twelve samples positive for *L. iners *(6 from normal flora and 6 from BV flora) to determine whether the abundance of *L. iners *was different in the two groups. The species-specific PCR for *L. iners *using serial dilutions of each sample revealed that both normal and BV flora contained 10^3 ^to 10^5 ^copies/*μ*l of *L. iners *and the median concentration was 10^4 ^copies/*μ*l for both.

As the presence of H_2_O_2_-producing lactobacilli in the vaginal fluid is associated with a reduced risk of BV [15,24] and because the concentration of H_2_O_2 _in the vaginal fluid is low in women with BV as compared with those with normal vaginal flora [21], the H_2_O_2_-producing ability of lactobacilli is thought to play a significant role in protecting the vaginal ecosystem from BV infection, although direct evidence to support this notion is lacking. Nearly all strains of *L. crispatus *and *L. jensenii *have been reported to produce H_2_O_2_, whereas only 9% of the strains of *L. iners *produce H_2_O_2 _[24]. The prevalences of *L. crispatus *and *L. jensenii *were significantly higher in the normal group than in the BV group and the detection rates of all BV-related bacteria were significantly higher in women with than in those without *L. iners *in this study. Although this observation is consistent with the notion that H_2_O_2_-producing ability of lactobacilli is important in protecting the vaginal ecosystem from BV infection, it remains to be determined whether these observations resulted from differences in H_2_O_2_-producing ability of these lactobacilli.

The newly proposed "BV-related bacteria," including BVAB2, *Megasphaera*, *Leptotrichia*, and *Eggerthella*-like bacterium, were all shown to be associated with BV in the present study, confirming the results of a recent study by Fredricks *et al*. [19]. However, the detection rates of these bacteria in women with BV were lower, while those in women with normal flora were similar to their results [19]. BVAB2 is cultivation-resistant, one of three bacteria (provisionally named BV-associated bacteria: BVAB1, BVAB2, and BVAB3) newly found to be highly specific for BV in the vagina of non-pregnant women [19], and not closely related to other bacteria as shown by comparison of 16S rDNA. In the present study, BVAB2 was present in 38.5% (5/13) and 3.1% (3/98) of women with BV and with normal vaginal flora, respectively, while Fredricks *et al*. reported these rates to be 88.9% (24/27) and 4.3% (2/46), respectively [19]. Similarly, detection rates of *Megasphaera *(69.2%),*Eggerthella*-like bacterium (53.8%), and *Leptotrichia *(53.8%) in women with BV in the present study were lower than those of 96.3%, 92.6%, and 85.2% reported by Fredricks *et al*. [19], while detection rates of *Megasphaera *(11.2%),*Eggerthella*-like bacterium (7.1%), and *Leptotrichia *(14.3%) in women with normal vaginal flora were comparable to their values of 8.7%, 8.7%, and 4.3%, respectively [19].

The results of the present study raised the possibility that the four BV-related bacteria were less prevalent in pregnant Japanese women with BV as compared with non-pregnant American women. However, the number of subjects with BV in the present study was too low to draw definitive conclusions about the prevalence of bacteria in different populations. Further studies using different demographic populations are needed to determine the roles of these BV-related bacteria in the pathogenesis of BV.

Twelve (92%) of 13 women with BV were positive for genus *Lactobacillus *by 16S rDNA PCR using the universal *Lactobacillus *primer, including 5 women with *L. iners*, one with both *L. iners *and *L. crispatus*, one with *L. crispatus*, and 5 with unspecified *Lactobacillus*. Of these 13 women, only one with *L. iners *was positive for *Lactobacillus *by general cultivation methods and positive for Gram-positive rods on Gram staining. These results suggested that many women with BV harbor genus *Lactobacillus *in the vagina and that the number of these lactobacilli colonizing the vagina is small. Further, as *L. iners *has been reported to require specialized blood agar media for isolation [29], the conventional culture method used in this study may have failed to reveal its colonization in the vagina.

## Conclusion

Our results suggested that BV-related bacteria, including BVAB2, *Megasphaera*, *Leptotrichia*, and *Eggerthella*-like bacterium, were associated with BV in pregnant Japanese women. The presence of *L. iners*, one of the common *Lactobacillus *species in the vagina, may be correlated with vaginal colonization by these BV-related bacteria. It remains to be determined whether BV-related bacteria cause BV or are common and abundant as a consequence of BV.

## Competing interests

The author(s) declare that they have no competing interests.

## Authors' contributions

The manuscript was written by RT. RT and TY contributed 16S rDNA-based bacterial identification. TY and IF supervised the microbiology laboratory work. TY and KC performed the statistical analyses. TY and MM provided clinical samples. HY, NS and HM critically reviewed the manuscript. All authors read and approved the final manuscript.

## Pre-publication history

The pre-publication history for this paper can be accessed here:



## References

[B1] Sobel JD (2000). Bacterial vaginosis. Annu Rev Med.

[B2] Wang J (2000). Bacterial vaginosis. Prim Care Update Ob Gyns.

[B3] Burton JP, Reid G (2002). Evaluation of the bacterial vaginal flora of 20 postmenopausal women by direct (Nugent score) and molecular (polymerase chain reaction and denaturing gradient gel electrophoresis) techniques. J Infect Dis.

[B4] Gravett MG, Hummel D, Eschenbach DA, Holmes KK (1986). Preterm labor associated with subclinical amniotic fluid infection and with bacterial vaginosis. Obstet Gynecol.

[B5] Krohn MA, Hillier SL, Lee ML, Rabe LK, Eschenbach DA (1991). Vaginal *Bacteroides *species are associated with an increased rate of preterm delivery among women in preterm labor. J Infect Dis.

[B6] McDonald HM, O'Loughlin JA, Jolley P, Vigneswaran R, McDonald PJ (1991). Vaginal infection and preterm labour. Br J Obstet Gynaecol.

[B7] Holst E, Goffeng AR, Andersch B (1994). Bacterial vaginosis and vaginal microorganisms in idiopathic premature labor and association with pregnancy outcome. J Clin Microbiol.

[B8] Hay PE, Lamont RF, Taylor-Robinson D, Morgan DJ, Ison C, Pearson J (1994). Abnormal bacterial colonisation of the genital tract and subsequent preterm delivery and late miscarriage. BMJ.

[B9] Hillier SL, Nugent RP, Eschenbach DA, Krohn MA, Gibbs RS, Martin DH, Cotch MF, Edelman R, Pastorek JG, Rao AV, McNellis D, Regan JA, Carey JC, Klebanoff MA (1995). Association between bacterial vaginosis and preterm delivery of a low-birth-weight infant. The Vaginal Infections and Prematurity Study Group. N Engl J Med.

[B10] Subtil D, Denoit V, Goueff FL, Husson MO, Trivier D, Puech F (2002). The role of bacterial vaginosis in preterm labor and preterm birth: a case-control study. Eur J Obstet Gynecol Reprod Biology.

[B11] Usui R, Ohkuchi A, Matsubara S, Izumi A, Watanabe T, Suzuki M, Minakami H (2002). Vaginal lactobacilli and preterm birth. J Perinat Med.

[B12] Leitich H, Bodner-Adler B, Brunbauer M, Kaider A, Egarter C, Husslein P (2003). Bacterial vaginosis as a risk factor for preterm delivery: A meta-analysis. Am J Obstet Gynecol.

[B13] Hillier SL, Martius J, Krohn M, Kiviat N, Holmes KK, Eschenbach DA (1988). A case-control study of chorioamnionic infection and histologic chorioamnionitis in prematurity. N Engl J Med.

[B14] Hill GB (1993). The microbiology of bacterial vaginosis. Am J Obstet Gynecol.

[B15] Hillier SL, Krohn MA, Rabe LK, Klebanoff SJ, Eschenbach DA (1993). The normal vaginal flora, H2O2-producing lactobacilli, and bacterial vaginosis in pregnant women. Clin Infec Dis.

[B16] Thorsen P, Jensen IP, Jeune B, Ebbesen N, Arpi M, Bremmelgaard A, Moller BR (1998). Few microorganisms associated with bacterial vaginosis may constitute the pathologic core: A population-based microbiologic study among 3596 pregnant women. Am J Obstet Gynecol.

[B17] Ferris MJ, Masztal A, Aldridge KE, Fortenberry JD, Fidel PL, Martin DH (2004). Association of *Atopobium vaginae*, a recently described metronidazole resistant anaerobe, with bacterial vaginosis. BMC Infect Dis.

[B18] Zhou X, Bent SJ, Schneider MG, Davis CC, Islam MR, Forney LJ (2004). Characterization of vaginal microbial communities in adult healthy women using cultivation-independent methods. Microbiology.

[B19] Fredricks DN, Fiedler TL, Marrazzo JM (2005). Molecular identification of bacteria associated with bacterial vaginosis. N Eng J Med.

[B20] Klebanoff SJ, Hillier SL, Eschenbach DA, Waltersdorph AM (1991). Control of the microbial flora of the vagina by H2O2-generating lactobacilli. J Infect Dis.

[B21] Al-Mushrif S, Jones BM (1998). A study of the prevalence of hydrogen peroxide generating *Lactobacilli *in bacterial vaginosis: the determination of H2O2 concentrations generated, *in vitro*, by isolated strains and the levels found in vaginal secretions of women with and without infection. J Obstet Gynaecol.

[B22] Onderdonk AB, Lee ML, Lieberman E, Delaney ML, Tuomala RE (2003). Quantitative microbiologic models for preterm delivery. J Clin Microbiol.

[B23] Wilks M, Wiggins R, Whiley A, Hennessy E, Warwick S, Porter H, Corfield A, Millar M (2004). Identification and H2O2 production of vaginal lactobacilli from pregnant women at high risk of preterm birth and relation with outcome. J Clin Microbiol.

[B24] Antonio MAD, Hawes SE, Hillier SL (1999). The identification of vaginal *Lactobacillus *species and the demographic and microbiologic characteristics of women colonized by these species. J Infect Dis.

[B25] Song YL, Kato N, Matsumiya Y, Liu CX, Kato H, Watanabe K (1999). Identification of and hydrogen peroxide production by fecal and vaginal lactobacilli isolated from Japanese women and newborn infants. J Clin Microbiol.

[B26] Vallor AC, Antonio MAD, Hawes SE, Hillier SL (2001). Factors associated with acquisition of, or persistent colonization by, vaginal lactobacilli: Role of hydrogen peroxide production. J Infect Dis.

[B27] Pavlova SI, Kilic AO, Kilic SS, So JS, Nader-Macias ME, Simoes JA, Tao L (2002). Genetic diversity of vaginal lactobacilli from women in different countries based on 16S rRNA gene sequences. J Appl Microbiol.

[B28] Vasquez A, Jakobsson T, Ahrne S, Forsum U, Molin G (2002). Vaginal *Lactobacillus *flora of healthy Swedish women. J Clin Microbiol.

[B29] Falsen E, Pascual C, Sjoden B, Ohlen M, Collins MD (1999). Phenotypic and phylogenetic characterization of a novel *Lactobacillus *species from human sources: description of *Lactobacillus *iners sp. nov. Int J Syst Bacteriol.

[B30] Burton JP, Cadieux PA, Reid G (2003). Improved understanding of the bacterial vaginal microbiota of women before and after probiotic instillation. Appl Environ Microbiol.

[B31] Antonio MAD, Rabe LK, Hillier SL (2005). Colonization of the rectum by *Lactobacillus *species and decreased risk of bacterial vaginosis. J Infect Dis.

[B32] Nugent RP, Krohn MA, Hillier SL (1991). Reliability of diagnosing bacterial vaginosis is improved by a standardized method of Gram stain interpretation. J Clin Microbiol.

[B33] Byun R, Nadkarni MA, Chhour KL, Martin FE, Jacques NA, Hunter N (2004). Quantitative analysis of diverse *Lactobacillus *species present in advanced dental caries. J Clin Microbiol.

[B34] Giorgi A, Torriani S, Dellaglio F, Bo G, Stola E, Bernuzzi L (1987). Identification of vaginal lactobacilli from asymptomatic women. Microbiologica.

